# Improved Parallel Magnertic Resonance Imaging reconstruction with Complex Proximal Support Vector Regression

**DOI:** 10.1038/s41598-018-33171-x

**Published:** 2018-10-10

**Authors:** Lin Xu, Qian Zheng, Tao Jiang

**Affiliations:** 1 Control Engineering College, Chengdu University of Information Technology, Chengdu, China; 2 Zhengzhou University of Light Industry, Zhengzhou, China

## Abstract

Generalized Auto-calibrating Partially Parallel Acquisitions (GRAPPA) has been widely used to reduce imaging time in Magnetic Resonance Imaging. GRAPPA synthesizes missing data by using a linear interpolation of neighboring measured data over all coils, thus accuracy of the interpolation weights fitting to the auto-calibrating signal data is crucial for the GRAPPA reconstruction. Conventional GRAPPA algorithms fitting the interpolation weights with a least squares solution are sensitive to interpolation window size. MKGRAPPA that estimates the interpolation weights with support vector machine reduces the sensitivity of the *k*-space reconstruction to interpolation window size, whereas it is computationally expensive. In this study, a robust GRAPPA reconstruction method is proposed that applies an extended proximal support vector regression (PSVR) to fit the interpolation weights with wavelet kernel mapping. Experimental results on *in vivo* MRI data show that the proposed PSVR-GRAPPA method visually improves overall quality compared to conventional GRAPPA methods, while it has faster reconstruction speed compared to MKGRAPPA.

## Introduction

Parallel imaging technology using multi-channel phased array coils is widely used to speed up Magnetic Resonance Imaging (MRI) scanning by acquiring only a fraction of *k*-space data. Alias-free image can be reconstructed from incomplete *k*-space data with various reconstruction algorithms, e.g., sensitivity encoding (SENSE), simultaneous acquisition of spatial harmonics (SMASH), generalized auto-calibrating partially parallel acquisition (GRAPPA) and their derivatives^1–7^. These reconstruction methods can be generally classified into image domain methods and *k*-space methods. The image domain methods^1,7^ unfold aliased signals in image with accurate coil sensitivity information. The *k*-space methods linearly join adjacent acquired data over all coils to interpolate the value of missing data where interpolation weights are estimated by fitting to a small number of additional acquired auto-calibration signals (ACS). The *k*-space methods are superior in cases where determination of the coil sensitivity is difficult, whereas conventional *k*-space methods suffer from low signal-noise-rate (SNR) of reconstructed image. This pitfall was resolved by GRAPPA that reconstructs individual coil image separately and then combines all coil images in a sum-of-squares (SOS) fashion^8^.

GRAPPA reconstruction accuracy strongly depends on the selection of interpolation window. Inadequate interpolation windows could cause either under-fitting or over-fitting of interpolation weights, which can lead to serious noise and artifacts in the reconstructed image. Methods in^9–11^ defined various kinds of error metrics to evaluate all potential interpolation windows and chose the interpolation window with minimal error to reconstruct image, whereas such methods just focus on the reconstruction error in calibration region. Nana *et al*.^12^ utilized the shift-invariance of interpolation weights in *k*-space to approximate the predication error by calculating the difference between acquired signals and their estimates obtained based on the interpolation of the missing data. Although this method can determine the optimal interpolation window, time-exhausted reconstruction of all acquired *k*-space data for each possible interpolation window limits its application on large number of coils data.

Although the optimal interpolation window can be determined with aforementioned methods under low reduce factors, the conventional GRAPPA reconstructions using linear interpolation perform poorly at high acceleration situations aroused by under-fitting of interpolation weights. Chang *et al*. proposed a nonlinear GRAPPA (NLGRAPPA) reconstruction^13^, which uses a truncated polynomial kernel to map the *k*-space data onto a higher dimension feature space. Despite providing a more accurate fitting on the spatial correlation of coils compared with linear GRAPPA, the nonlinear transformation makes NLGRAPPA reconstruction more sensitive to interpolation windows. KerNL^14^ introduced kernel tricks to represent the general nonlinear relationship between acquired and missing *k*-space data that improves both image quality and computation efficiency at high reduction factors. More recently, MKGRAPPA^15^ reformulated estimating the optimal interpolation weights as a multi-kernel learning problem that can reduce the sensitivity of the *k*-space reconstruction to interpolation windows. Nevertheless, the MKGRAPPA is computationally expensive due to the involved semi-infinite linear programming.

In this study, we theoretically analyze errors in GRAPPA reconstruction from the perspective of structure risk minimization (SRM) principle^16^ that can achieve favorable compromise between fitting error and complexity of fitting function for regression problem. We thereby develop a robust GRAPPA reconstruction method that estimates the interpolation weights by proximal support vector machine (PSVR)^17^. PSVR is a advantaged version of support vector machine (SVM)^16^ developed from the SRM principle. PSVR has looser constraints than does SVM, with comparable performance and much lower computational cost. Experimental results of *in vivo* brain imaging are provided to demonstrate the performance of the proposed method named “PSVR-GRAPPA” compared with the GRAPPA and MKGRAPPA methods.

The rest of this paper is organized as follow: Section 2 reviews the theory of GRAPPA algorithm and SRM, then describes the details of proposed method which applies an extended PSVR to fit the interpolation weights with wavelet kernel mapping; Section 3 applies the proposed method *in vivo* experiments; Section 4 gives the conclusions and future work to be done.

## Methods

### Review of GRAPPA

GRAPPA estimates the missing *k*-space data in each coil by a linear combination of its nearby acquired data over all coils, which can be mathematically represented as

1 $$sl(kx,ky+m{\rm{\Delta }}ky)=\sum _{j=1}^{L}\,\sum _{{h=H}_{{\rm{1}}}}^{{H}_{2}}\,\sum _{t={N}_{1}}^{{N}_{2}}{w}_{l,m}(j,h,t){s}_{j}\,(kx+h\cdot {\rm{\Delta }}kx,ky+t\cdot R\cdot {\rm{\Delta }}ky)$$

where *s* represents the *k*-space signal and *w* denotes the interpolation weights, (*k*_*x*_*, k*_*y*_) are *k*-space coordinates along the phase encoding (PE) and frequency encoding (FE) directions, respectively. Δ*k*_*x*_ and Δ*k*_*y*_ are sampling intervals along the PE and FE directions, respectively. In Eq. (1), *j* and *l* represent coil indexes, *L* is the number of coils in the array, *m* is the offset from the acquired data, *N*_1_ and *N*_2_ define the lower and upper bounds of the interpolation window along the PE direction, *H*_1_ and *H*_2_ define the left and right bounds of the interpolation window along the FE direction, respectively.

**Figure 1:**
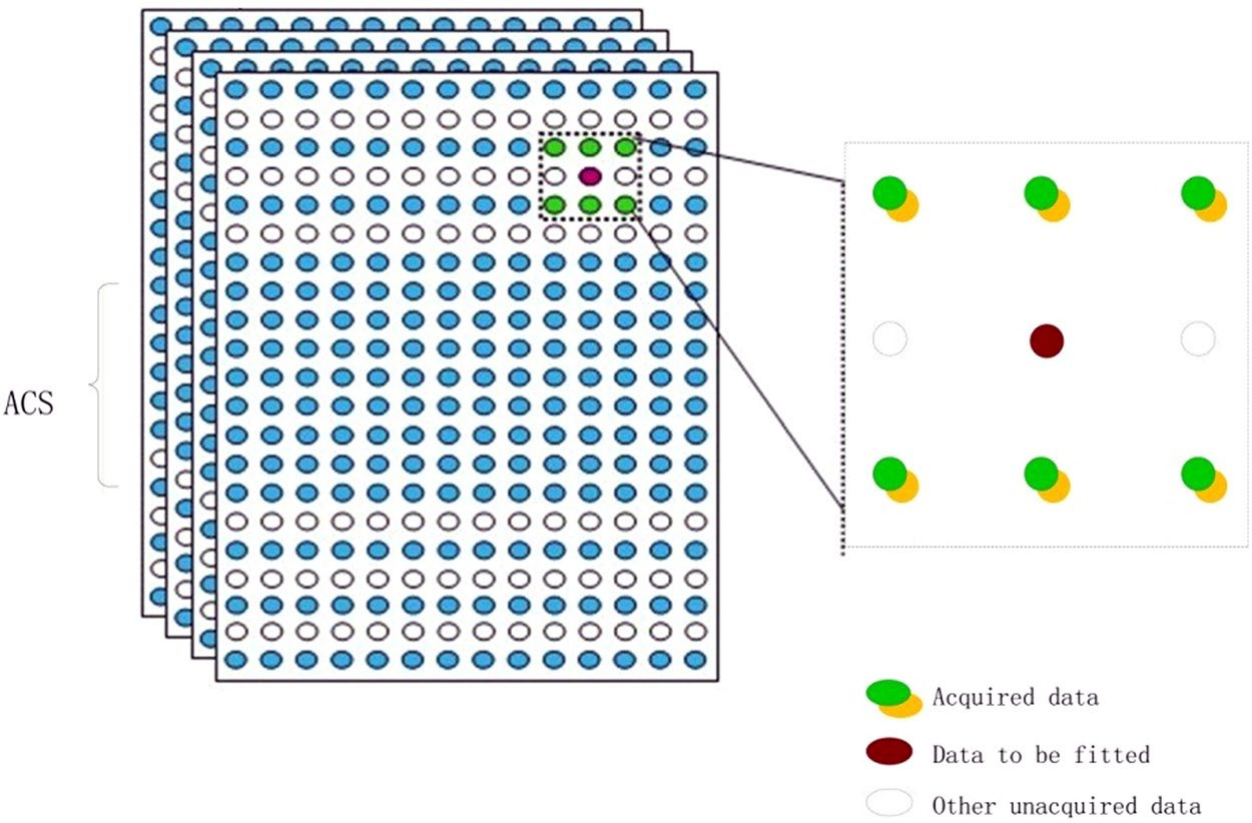
Schematic description of GRAPPA.

As shown in Fig. 1, the central region of *k*-space is fully sampled to calibrate the interpolation weights *w*. A linear system with knowing input and output is constructed as follows,

2 $$y=Aw+\varepsilon $$

where *y* is a vector constituted of all target data, and *A* is an $\ell$*M matrix composed of the interpolation source data supposing that there are *M* interpolation source data points in the interpolation window and $\ell$ target data points to be interpolated.

### SRM model of GRAPPA

In general, conventional GRAPPA reconstruction methods utilize the least squares method to estimate the interpolation coefficients *w*, which minimizes the following empirical error,

3 $${R}_{emp}(w)={{\sum }_{i=1}^{\ell }({y}_{i}-{\sum }_{k=1}^{M}{w}_{k}{a}_{i}^{k})}^{2}={\Vert {y}_{i}-{\sum }_{k=1}^{M}{w}_{k}{a}_{i}^{k}\Vert }_{2}$$

Equation (3) indicates that *R*_emp_(*w*) decreases with the increasing of interpolation window size, and *R*_emp_(*w*) gets close to zero when *M* equals to *l*. Nonetheless, total error would not be minimal as interpolation window is oversized. Previous studies^11,12^ indicate that the GRAPPA reconstruction error would increase as the interpolation window size increases if the latter reaches a threshold. Therefore, both interpolation weights *w* and interpolation window size *M* need to be optimally determined to obtain a perfect reconstruction.

**Figure 2:**
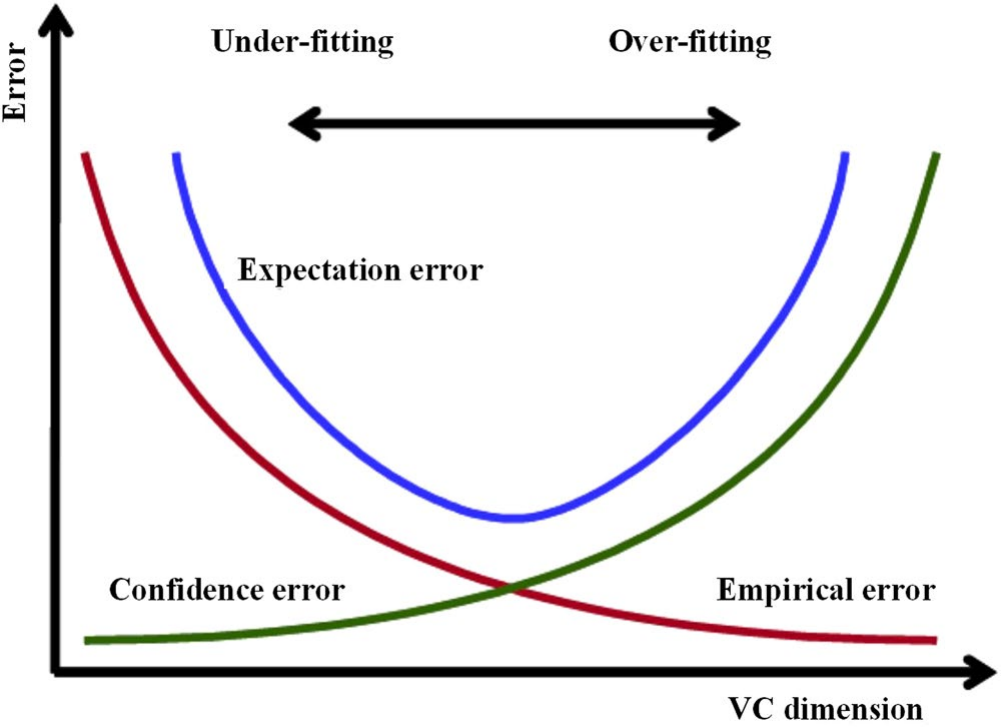
The expectation error is the sum of the empirical error and the confidence error. The empirical error is decreased with the VC dimension, while the confidence error is increased.

GRAPPA reconstruction can be viewed as a supervised learning problem. According to the capacity concept of Vapnik-Chervonenkis (VC) theory^16^, the total error of supervised learning problem can be described as expectation error *R*(*w*), which can be represented as follows,

4 $${\rm{R}}(w)\le {{\rm{R}}}_{{\rm{emp}}}(w)+{\rm{\Phi }}(h/\ell )$$

where *R*_emp_(*w*) is the empirical error decreased as *h* increases, and $\Phi (h/\ell )$ is confidence error. *h* is VC dimension indicating the complexity of interpolation weights. The dimension of interpolation weights *w* is a good estimation of VC dimension for linear interpolation weights, thus a larger interpolation window indicates a higher VC dimension. Equation (4) shows that increasing the interpolation window size can reduce the empirical error while increase the confidence error. As shown in Fig. 2, the expectation error is an “U”-shape function of VC dimension. Consequently, the total expectation error will increase when the interpolation window size increases after a certain value.

Actually, the least squares solution only minimizes *R*_emp_(*w*) but not *R*(*w*), which is the optimal solution in the sense that $\ell \to +\infty$, i.e., $\Phi ({\rm{h}}/\ell )\to 0$. Without considering the confidence error, the interpolation weights fitted with the least squares method have poor generalization ability that the fitted model adapts properly to new data.

### Proposed PSVR-GRAPPA

It is well-known that superior generalization performance can be obtained from SVM and more importantly, the performance does not depend on the dimensionality of the input data^16^. Therefore, the SVM could effectively address the aforementioned problem in the GRAPPA reconstruction. Unfortunately, the original SVM that solves the constrained quadratic programming (QP) problem with linear constraints has a time complexity O(*N*^3^)^18^. PSVR is an alternate formulation of SVM regression that dramatically reduces the computation. Consequently, a complex PSVR for GRAPPA is proposed to reduce the sensitivity of interpolation window size in GRAPPA reconstruction.

### Complex PSVR for GRAPPA

GRAPPA employs an unbiased interpolation weights to approximate the relation of interpolation source and target data, whereas recent work^13^ indicated that the bias drastically increases as the noise increases at high accelerations. For this reason, we added a bias variable “*b*” into the cost function to improve fitting accuracy. Hence, interpolation weights can be solved with the following optimization problem:

5 $$\begin{array}{c}J=\,{\rm{\min }}\,\frac{1}{2}({w}^{H}w+{{\rm{b}}}^{H}{\rm{b}})+\frac{C}{2}\sum _{k=1}^{l}{{\eta }_{k}}^{H}{\eta }_{k}\\ {\rm{s}}{\rm{.t}}\,{y}_{k}-{w}^{H}\phi ({x}_{k})-b={\eta }_{k},k=1,\mathrm{...},l\end{array}$$

where *w* and *b* are coefficients of interpolation weights, *C* is a punishment factor to balance the training error rate and the complexity of the model. *η*_*κ*_ is a slack variable for residual term. The superscript H denotes the operation of Hermitian Transpose. *φ*(*x*_*κ*_) is a vector containing the source data to interpolate target data *y*_*κ*_. In Eq. (5), the *w*^*H*^*w* is an estimate of VC dimension of interpolation weights and *η*_*κ*_^*H*^*η*_*κ*_ represents individual empirical error, thereby minimizing the sum of such two terms can obtain an optimal solution with minimal expectation error. Theoretically, the minimal expectation error can give the best reconstruction quality given the training samples and interpolation window size. However, it is worth to note that inappropriate determination parameter would discount the performance of minimal expectation error. A detailed parameters optimization will be given in the below subsection.

Considering that the *k*-space data are complex value while the original PSVR was proposed at real space, we define the operation

6 $$L({\varepsilon }_{n})=L(\Re \{{\varepsilon }_{n}\})+L(\Im \{{\varepsilon }_{n}\})$$

where $\Re \{\}$ and $\Im \{\}$ denote the operations of taking the real part and imaginary part of complex data, respectively. Consequently, problem Eq. (5) can be stated as:

7 $$\begin{array}{rcl}J & = & {\rm{\min }}\,\frac{1}{2}({w}^{H}w+{{\rm{b}}}^{H}{\rm{b}})+\frac{C}{2}\sum _{k=1}^{l}({{\xi }_{k}}^{2}+{{\zeta }_{k}}^{2})\\  &  & {\rm{s}}{\rm{.t}}\,\Re ({y}_{k}-{w}^{H}\phi ({x}_{k})-b)={\xi }_{k},k=1,\mathrm{...},l\\  &  & \Im ({y}_{k}-{w}^{H}\phi ({x}_{k})-b)={\zeta }_{k},k=1,\mathrm{...},l\end{array}$$

where slack variables *ξ*, ζ are introduced for both real and imaginary residual terms. The dual problem of Eq. (7) is obtained by introducing Lagrange multipliers *α* and *β*:

8 $$\begin{array}{rcl}L & = & {\rm{\min }}\,\frac{1}{2}({w}^{H}w+{{\rm{b}}}^{H}{\rm{b}})+\frac{C}{2}\sum _{k=1}^{l}({{\xi }_{k}}^{2}+{{\zeta }_{k}}^{2})\\  &  & +\,\sum _{k=1}^{l}{\alpha }_{k}\Re [{w}^{H}\phi ({x}_{k})+b+{\xi }_{k}-{y}_{k}]\\  &  & +\,\sum _{k=1}^{l}{\beta }_{k}\Im [{w}^{H}\phi ({x}_{k})+b+{\xi }_{k}-{y}_{k}]\end{array}$$

From the Karush-Kuhn-Tucker (KKT) conditions for optimality, we find:

9 $$\begin{array}{rcl}\frac{\partial L}{\partial w} & = & 0\to w=-\,\sum _{k=1}^{l}{\alpha }_{k}\Re (\phi ({x}_{k}))-\sum _{k=1}^{l}{\beta }_{k}\Im (\phi ({x}_{k}))\\ \frac{\partial L}{\partial b} & = & 0\to \sum _{k=1}^{l}({\alpha }_{k}+j\cdot {\beta }_{k})=-\,b\\ \frac{\partial L}{\partial \xi } & = & 0\to {\alpha }_{k}=C\cdot {\xi }_{k},k=1,\mathrm{...},l\\ \frac{\partial L}{\partial \zeta } & = & 0\to {\beta }_{k}=C\cdot {\zeta }_{k},k=1,\mathrm{...},l\\ \frac{\partial L}{\partial \alpha } & = & 0\to \Re [{w}^{H}\phi ({x}_{k})+b+{\xi }_{k}-{y}_{k}]=0\\ \frac{\partial L}{\partial \beta } & = & 0\to \Im [{w}^{H}\phi ({x}_{k})+b+{\xi }_{k}-{y}_{k}]=0\end{array}$$

After elimination of the variables *w*, *ξ* and ζ, one can derive the following linear system:

10 $$[\begin{array}{cc}{\Omega }_{r}+{C}^{-1}I-{\overrightarrow{1}}_{v}^{T}{\overrightarrow{1}}_{v} & 0\\ 0 & {\Omega }_{i}+{C}^{-1}I-{\overrightarrow{1}}_{u}^{T}{\overrightarrow{1}}_{u}\end{array}]\,[\begin{array}{c}\alpha \\ \beta \end{array}]=[\begin{array}{c}\Re (y)\\ \Im (y)\end{array}]$$

where *I* is an identity matrix,$\overrightarrow{1}={[1;\mathrm{...};1]}^{{\rm{T}}}$, *Ω*_*r*_ and *Ω*_*i*_ are Gramm matrices wherein ${\Omega }_{kl}=K({x}_{k},{x}_{l}),k=1,\mathrm{...},l$, and *K*(*x*_*k*_, *x*_*l*_) is a kernel function that will be described in the below subsection.

Lagrange multipliers *α* and *β* can be solved in Eq. (10) with ACS data, then other missing data can be predicted as follows,

11 $$\begin{array}{rcl}f(m) & = & {w}^{H}\phi (m,x)+b=\sum _{k=1}^{l}{\alpha }_{k}K(\Re (m^{\prime} ),\Re ({x}_{k}))\\  &  & +\,{j}^{\ast }\sum _{k=1}^{l}{\beta }_{k}K(\Im (m^{\prime} ),\Im ({x}_{k}))+b\end{array}$$

where vector *x* consists of interpolation source data in ACS, and *m*′ denotes the surrounding acquired data of missing data *m*, *j* is the square root of −1. The bias term *b* can be computed as follows,

$$b=-\,\sum _{k=1}^{l}({\alpha }_{k}+j\cdot {\beta }_{k})$$

## Experments and Results

### Data acquisition

The proposed method was evaluated on two sets of *in vivo* human brain data (axial and sagittal) acquired using an SE pulse sequence (TE/TR = 14/400 ms, 33.3 kHz bandwidth, 256 × 256 pixels, FOV = 240*240 mm^2^) on a 1.5 T scanner (Siemens Healthcare, Erlangen, Germany) with an 8-channel head coils. The data were fully sampled and later decimated in the PE direction by various factors to mimic parallel imaging acquisition procedure. All *in vivo* data were collected from healthy adult human volunteers with written informed consent from the participants in accordance with policies of the Institutional Review Board(IRB). All experimental protocols were approved by the IRB of University of Electronic Science and Technology of China (Chengdu, China).

### Parameters optimization

The punishment factor *C* needs to be optimally tuned in the proposed method. Although global or local optimization techniques such as genetic or gradient algorithms can obtain an approximately optimal parameter, such time-consuming methods may not be proper in the applications of real-time requirement. In this work, a heuristics selection algorithm^19^ is utilized for the determining of punishment factor *C*:

12 $$C=\,\max (|\bar{y}+3\sigma |,|\bar{y}-3\sigma |)$$

where $\bar{{\rm{y}}}$ and *σ* are mean and standard deviation of interpolation target *k*-space data in ACS region, respectively. Consequently, the parameter *C* was adaptively computed for each reconstruction task.

The kernel mapping is the basic characteristic for SVM. One can flexibly choose appropriate kernels for various applications. Nonetheless, it is intractable to choose the best kernel in practical application. Therefore, the performances of common used kernels were investigated with a set of sagittal data in this study. GRAPPA and PSVR-GRAPPA with different kernels were compared under the same sampling condition: *R* is 5 and 40 ACS lines were used to calibrate the interpolation weights. These kernels include:

Polynomial: $K(x,y)={(x\cdot y+\theta )}^{d}$, where *θ* and *d* are positive integers to define the constant term and order, respectively.

RBF: $K(x,y)=\exp (\,-\,{\Vert x-y\Vert }^{2}\,/\,{\gamma }^{2})$, where *γ* is a parameter to turn the width of RBF kernel.

Wavelet kernel: $K(x,y)=\prod _{i=1}^{N}h(\frac{{x}_{i}-{y}_{i}}{q})$ where $h(x)=\,\cos (1.75x)\exp (-\frac{{x}^{2}}{2})$.

The polynomial kernel with 2-order and *θ* = 1, the width of RBF kernel *γ* was chosen followed the work^18^: $\gamma =\frac{{d}_{\max }}{\sqrt{2l}}$, where *d*_*max*_ is the maximum distances between all training data. Based upon a number of empirical comparisons, we found that the following equation works well for estimating a desirable scale factor for wavelet kernel:

13 $$q={d}_{\max }\,\mathrm{log}\,l$$

### Image reconstruction

Reconstructions were performed on a desktop personal computer using MATLAB (The Mathworks, Natick, MA, USA). A quantitative assessment of reconstruction performance was performed by computing the NMSE and PSNR of reconstructed image:

$$\begin{array}{c}NMSE=\sqrt{\sum _{i=1}^{N}\sum _{j=1}^{N}{|{I}_{i,j}^{recon}-{I}_{i,j}^{ref}|}^{2}/\sum _{i=1}^{N}\sum _{j=1}^{N}{|{I}_{i,j}^{ref}|}^{2}}\\ {\rm{PSNR}}=10\times \,\mathrm{log}(\mathrm{255}\times \mathrm{255}/NMSE)\end{array}$$

where ${I}_{i,j}^{ref}$ is the result of non-accelerated scenario, *i* and *j* are the indices of image pixel along PE and FE directions, respectively.

The effect of interpolation window size on reconstruction quality was investigated with the sagittal data. As reduce factor *R* increased from 2 to 4, the images were reconstructed by GRAPPA and PSVR-GRAPPA with varying interpolation windows, respectively. The interpolation window size along the PE direction was fixed to 2, and that along the FE direction was varied from 1 to 15. ACS lines were adopted with 12, 24 and 40 for *R* of 2, 3 and 4, respectively. To qualitatively analysis the effect of interpolation window size on reconstruction quality, the axial brain dataset was reconstructed by GRAPPA and PSVR-GRAPPA with three kinds of interpolation windows: 4*3, 2*7, 4*15. The sampling parameters: *R* = 3, ACS lines = 24.

The reconstruction quality of PSVR-GRAPPA with wavelet kernel mapping was compared against those of GRAPPA and MKGRAPPA with the sagittal brain dataset. All methods reconstructed in the entire *k*-space with an interpolation window size of 2*9. The sampling parameters: *R* = 4, ACS lines = 40. The full *k*-space data were reconstructed by 2-D Inverse Fast Fourier Transform (IFFT) for each coil, and then combined all coil images in the SOS method to give the reference image.

### Results

**Figure 3:**
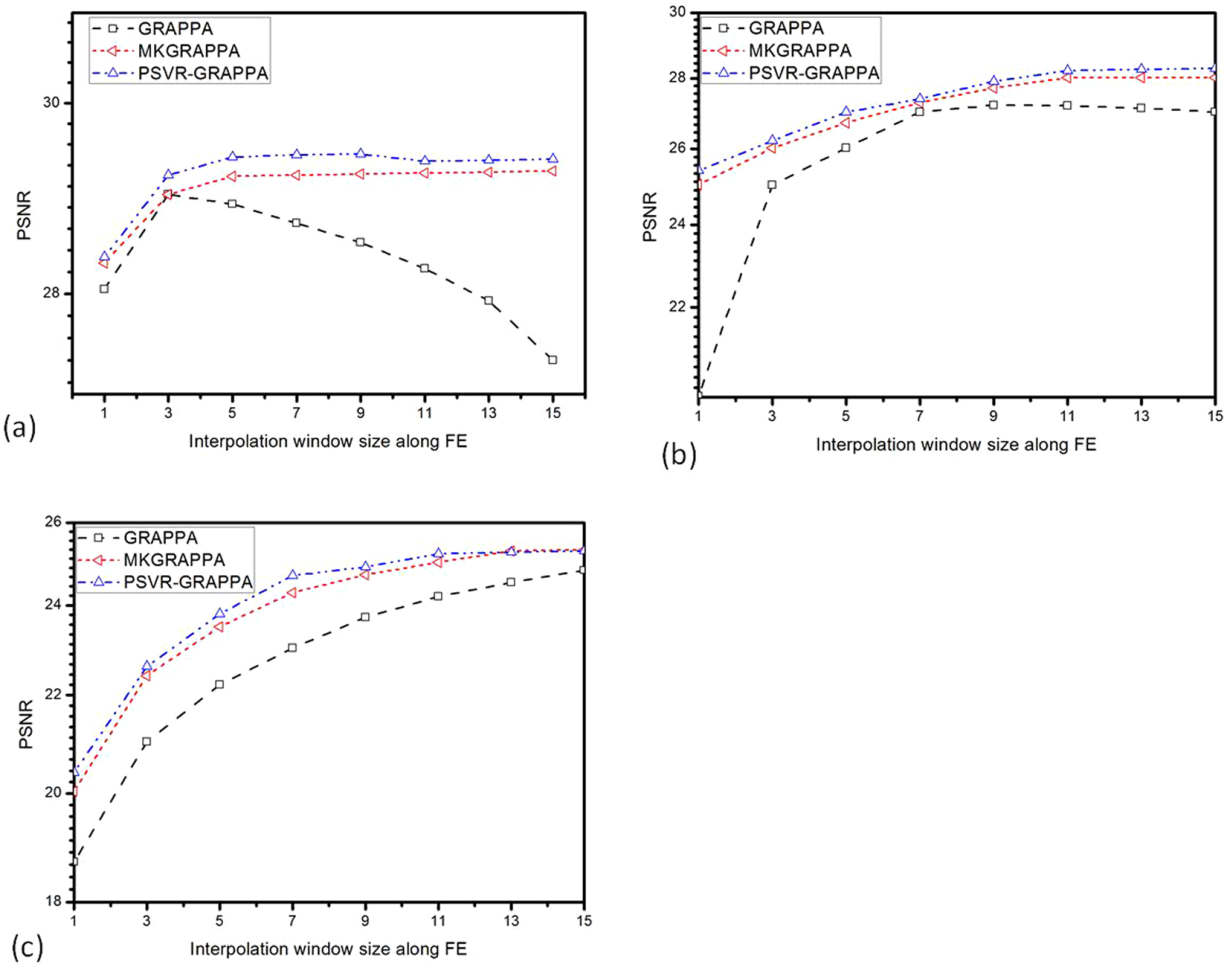
Plots of PSNR of brain data vs. interpolation window size along the FE direction for different sampling conditions: (**a**) *R* = 2, ACS lines = 12; (**b**) *R* = 3, ACS lines = 24; and (**c**) *R* = 4, ACS lines = 40.

**Figure 4:**
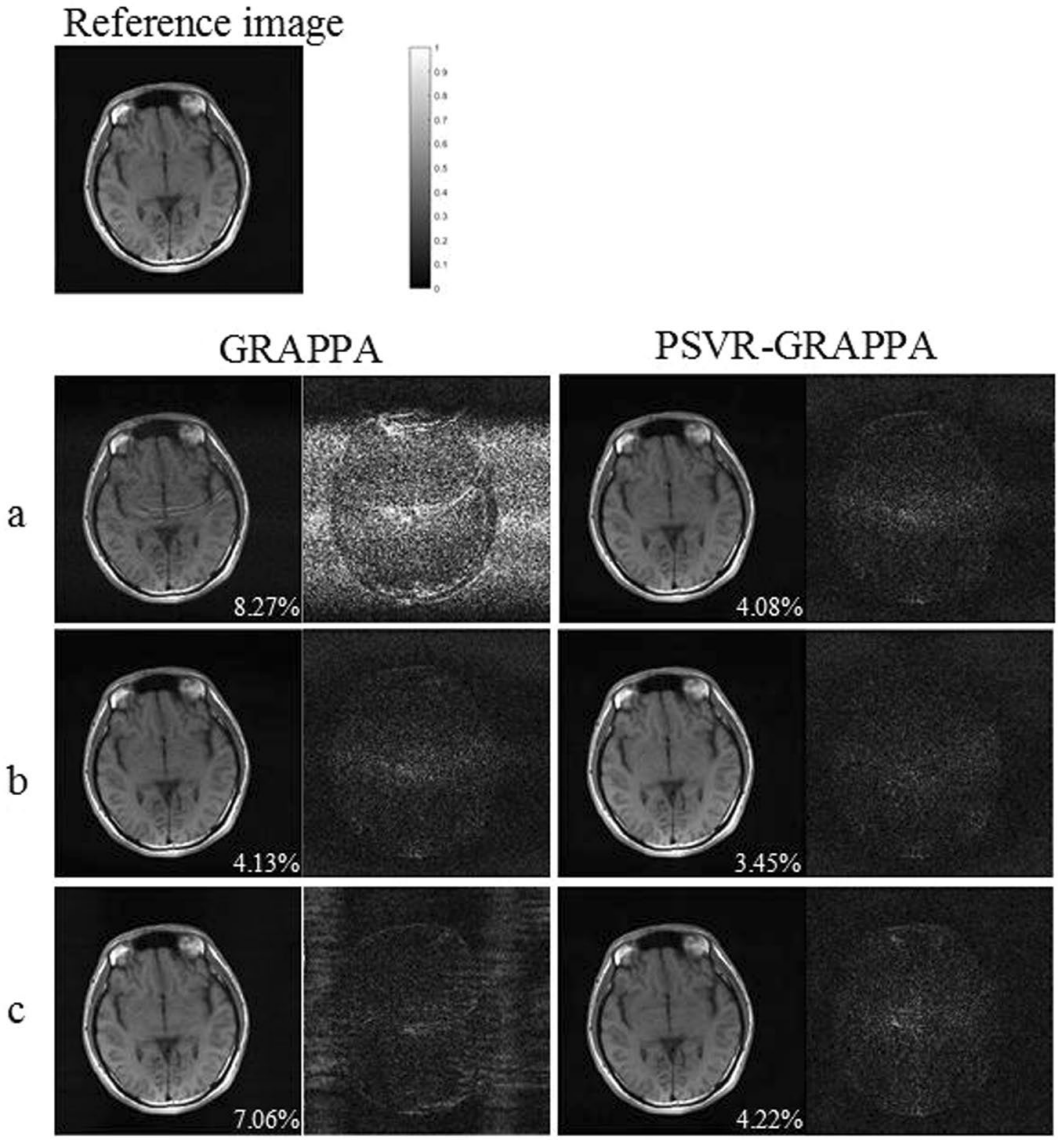
Axial brain images reconstructed by GRAPPA(left) and PSVR-GRAPPA (right) under sampling parameters: *R* = 3 with 24 ACS lines. Both methods were reconstructed in the entire *k*-space with interpolation windows: 4*3(**a**), 2*7 (**b**) and 4*15 (**c**). Nonaccelerated image is used as reference (top). To the right of each reconstructed image its absolute error with the nonaccelerated image is shown.

Figure 3 presents plots of variation of PSNR as increasing of interpolation window size in GRAPPA, MKGRAPPA and PSVR-GRAPPA at *R* of 2 (Fig. 3a), 3 (Fig. 3b) and 4 (Fig. 3c). Black lines represent the results of GRAPPA, red lines represent the results of GRAPPA, and blue lines indicate the results of PSVR-GRAPPA. Totally PSVR-GRAPPA reconstructed images have highest PSNRs among the three methods. All methods have low PSNRs when the interpolation window size is small regardless to the *R*. This phenomenon can be interpreted with the under-fitting of interpolation weights aroused by the small size of interpolation window. Therefore, the PSNR can be increased by increasing the interpolation window size. In Fig. 3a,b, the increasing of the interpolation window will decrease PSNR once the interpolation window size reaches a certain threshold, which is caused by the over-fitting of interpolation weights. At high accelerations (Fig. 3c), the linear model cannot catch sufficiently the complexity of spatial information of coils in *k*-space since the interpolation target data are far away from the interpolation source data, which results an under-fitting of interpolation weights. As a result, the fitting error that decreases as VC dimension *h* increases grows to be the dominating error. Hence, the PSNRs of all three methods increased with the increasing of the interpolation window size in Fig. 3c. Nonetheless, the PSNRs of MKGRAPPA and the proposed method increase faster than that of GRAPPA because that the nonlinear kernel has a higher *h* than linear model does. Although Fig. 3 shows that the reconstruction performance of all methods can be affected with interpolation window size along FE, MKGRAPPA and PSVR-GRAPPA based on the structural risk minimization theory are less sensitive to the changes of interpolation window than GRAPPA.

Figure 4 shows axial brain images reconstructed by GRAPPA and PSVR-GRAPPA with interpolation windows: 4*3(Fig. 4a), 2*7(Fig. 4b) and 4*15(Fig. 4c). The absolute error image is show on the right of the reconstruction result, respectively. Serious aliasing artifacts and amplified noise can be seen in the image reconstructed by GRAPPA with the interpolation window of 4*3, which results from the under-fitting of interpolation weights. Aliasing artifacts and amplified noise are significantly alleviated in the image reconstructed by GRAPPA with the interpolation window of 2*7. The image given by GRAPPA with the interpolation window of 4*15 has less noise at the cost of increased artifacts arising from the over-fitting of interpolation weights due to the over-sized interpolation window. Images reconstructed by PSVR-GRAPPA has no obvious aliasing artifacts and noise. Furthermore, less difference can be found among images reconstructed by PSVR-GRAPPA with such three kinds of interpolation windows. Quantitatively, PSVR-GRAPPA consistently yields smaller NMSEs than GRAPPA.

**Figure 5:**
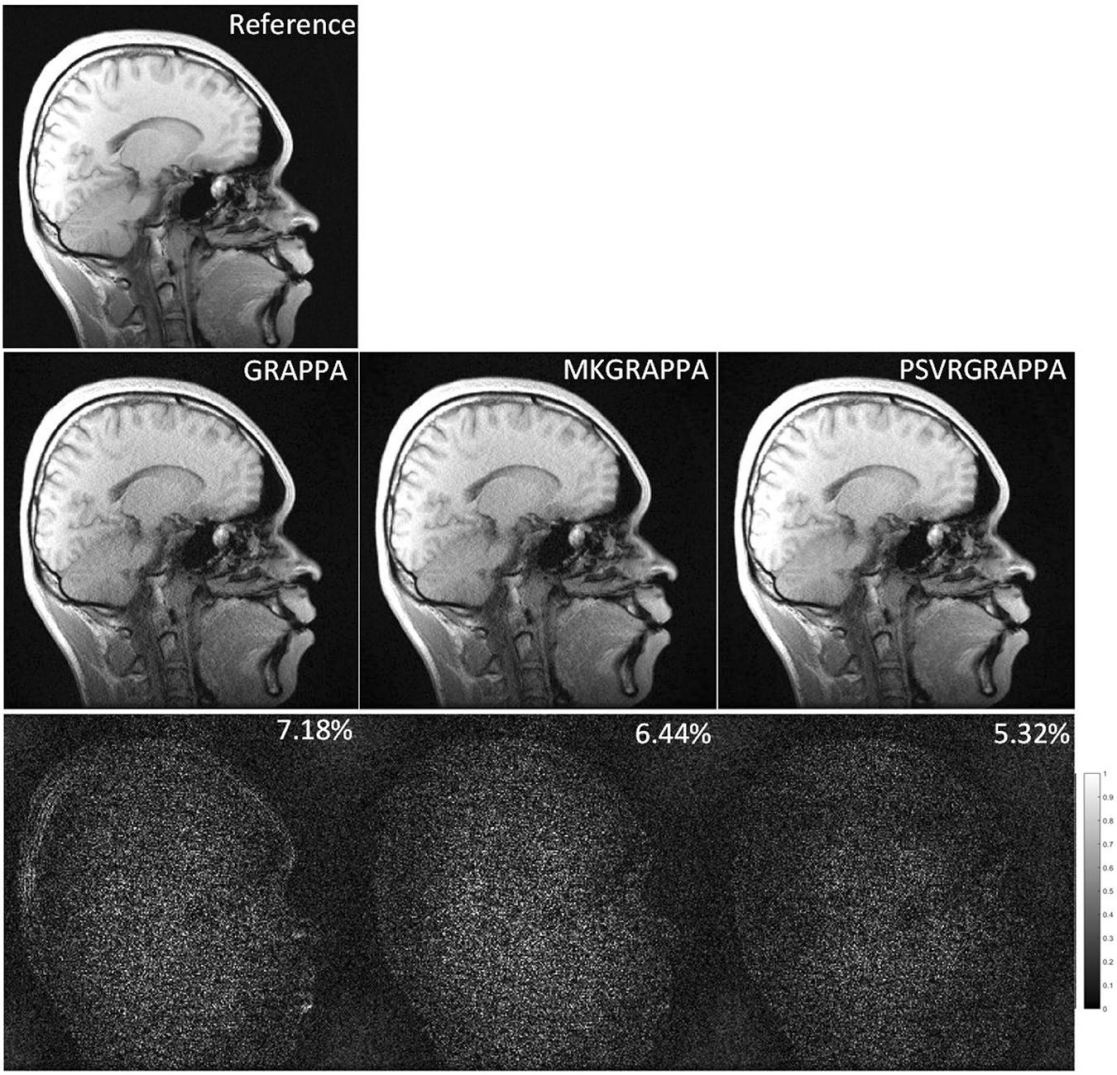
Sagittal brain images reconstructed by GRAPPA(left), MKGRAPPA(middle), and PSVR-GRAPPA (right) under sampling parameters: *R* = 4 with 40 ACS lines. All methods were reconstructed in the entire *k*-space with an interpolation window of 2*9. Nonaccelerated image is used as reference (top). To the bottom of each reconstructed image its absolute error with the nonaccelerated image is shown.

Figure 5 compares GRAPPA, MKGRAPPA, and PSVR- GRAPPA with an *R* of 4 using the sagittal brain dataset. The images reconstructed by GRAPPA and MKGRAPPA contain noticeable aliasing artifacts, which are significantly alleviated by PSVR-GRAPPA. Quantitatively, the NMSE of the PSVR-GRAPPA reconstruction is 5.32%, which is lower than those of GRAPPA (7.18%) and MKGRAPPA (6.44%) reconstructions.

Table 1 lists the time consumption of three methods *in vivo* studies. MKGRAPPA has the largest time consumption owing to the semi-infinite linear programming. Nevertheless, the proposed PSVR-GRAPPA is slightly slower than GRAPPA.

**Table 1:** Comparison of time consumption between GRAPPA, MKGRAPPA and PSVR-GRAPPA(sec).

	*R* = 3 ACS = 24	*R* = 4 ACS = 40	*R* = 5 ACS = 60
GRAPPA	11	41	245
NLGRAPPA	17	103	348
MKGRAPPA	14	50	279

**Figure 6:**
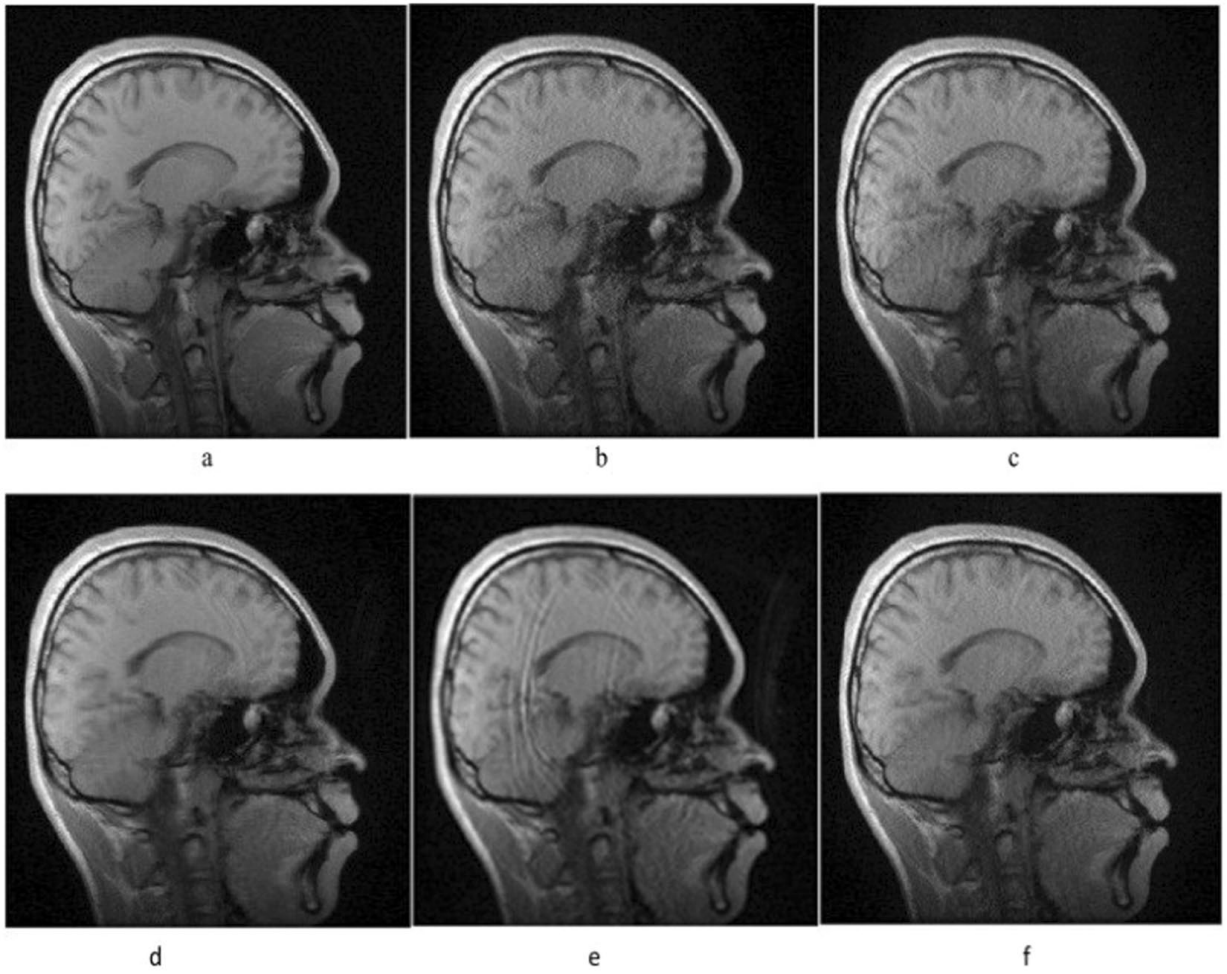
Sagittal brain images reconstructed by IFFT with full *k*-space data (**a**), GRAPPA method (**b**), and PSVR-GRAPPA with linear kernel (**c**), Polynomial kernel (**d**), RBF(**e**), wavelet kernel (**f**).

Figure 6 shows images reconstructed by different kernels. Figure 6a is the reference image reconstructed with full *k*-space data, other result images are reconstructed by GRAPPA method (Fig. 6b), and PSVR-GRAPPA with linear kernel (Fig. 6c), polynomial kernel (Fig. 6d), RBF kernel (Fig. 6e), wavelet kernel (Fig. 6f), respectively. The image reconstructed by linear kernel has similar noise level as GRAPPA reconstructed image, the noise is significantly reduced in the image resulted by polynomial kernel at the cost of blurs and artifacts, and the artifacts is severe in the image of RBF kernel reconstructed image. The noise is suppressed in the image reconstructed by wavelet kernel without visible artifacts, which looks closer to the reference image (Fig. 6a). In theory, wavelet kernel can approximate arbitrary functions that can give better outcomes than other kernels^20^.

## Conclusions

This study introduces a novel GRAPPA reconstruction method based on proximal support vector regression. The proposed PSVR-GRAPPA method has the advantage of adaptively balancing under- fitting and over-fitting of interpolation weights without significant increase in computational load. The *in vivo* experiments have demonstrated the proposed approach can significantly reduce the noise and aliasing artifacts in GRAPPA reconstruction and the reconstruction performance is insensitive to the interpolation window configuration. Compared with MKGRAPPA, although PSVR-GRAPPA gives implicit improvement on image quality, the latter has less computational load than the former.
